# Supplementary material for: Exactly solving the Kitaev chain and generating Majorana-zero-modes out of noisy qubits
Source: Sci Rep. 2022 Nov 18;12:19882. doi: 10.1038/s41598-022-24341-z (PMC9674617; doi:10.1038/s41598-022-24341-z)
Supplement: Supplementary file 1 — Supplementary Information. [file 41598_2022_24341_MOESM1_ESM.pdf]

Supplementary Material: Exactly solving the Kitaev chain and generating Majorana-zero-modes out of noisy qubits

Marko J. Rančić*

Address TotalEnergies, Tour Coupole La Défense, 2 Pl. Jean Millier, 92078 Paris

S1 - PREPARING EIGENSTATES OF QUADRATIC HAMILTONIANS ON A QUANTUM COMPUTER

In this Section I will briefly outline the procedure for obtaining the ground state of quadratic Hamiltonians relying heavily on steps described in Appendix A and Section IV of Ref. [1]. I will try to use the same notation and follow exactly the same steps. The reader is refereed to the original study for a more detailed discussion. This procedure is implemented in Google's Quantum AI Cirq in an exact numerical fashion [2]. Afterwards, The circuit obtained in Google's Quantum AI Cirq is converted to a Qiskit form and the code for that is given in 10.5281/zenodo.6323467.

The procedure is as follows:

1. Find A the Majorana representation of a general quadratic Hamiltonian $\mathcal{H} = \frac{i}{2}\mathbf{f}^T A\mathbf{f}$.

2. Bring the matrix A into the Schur form and permute indices such that the upper block ε is diagonal with eigenvalues in increasing order

$$\text{perm}(\text{Schur}(A)) = RAR^T = \begin{bmatrix} 0 & \varepsilon \\ -\varepsilon & 0 \end{bmatrix}. \tag{S1}$$

3. Obtain $W = \Omega^\dagger R\Omega$, where

$$\Omega = \frac{1}{2}\begin{pmatrix} \mathbb{1} & \mathbb{1} \\ i\mathbb{1} & -i\mathbb{1} \end{pmatrix}. \tag{S2}$$

The form of W is in general

$$W = \begin{bmatrix} W_1^* & W_2^* \\ W_2 & W_1 \end{bmatrix}. \tag{S3}$$

4. Find such unitary transformation U for which $VW_LU = [0\,\mathbb{1}]$, where V is a general unitary matrix, $W_L = [W_1^* W_2^*]$.

5. U can be decomposed into products of X gates on the last qubit and a combination of $RYXXY$ and single qubit Z gates - and it is a representation of a unitary operator which diagonalizes a general quadratic Hamiltonian.

S2 - THE WINDING NUMBER - A TOPOLOGICAL INVARIANT FOR A FINITE KITAEV CHAIN

In this Section I will give an answer to the question of the boundary of the topological phase of finite Kiteav chains. To paraphrase it more specifically and in a more simple manner: when $t = -\Delta$ and n is finite, for which value of μ does a topological-non-topological transition occur in the Kitaev Hamiltonian?

The starting point is the derivation is n-site Kitaev Hamiltonian

$$H = \sum_{k=1,n} \mu_k c_k^\dagger c_k - \sum_{\langle kj \rangle} \left(t_{kj} c_k^\dagger c_j - \Delta_{kj} c_k^\dagger c_j^\dagger + H.c. \right). \tag{S4}$$

Now I perform a discrete Fourier transform of Eq. (S4) by setting $c_k^\dagger = \frac{1}{\sqrt{n}}\sum_K \exp(-ikK)c_K^\dagger$ and $c_k = \frac{1}{\sqrt{n}}\sum_K \exp(ikK)c_K$. Here the capital K denotes the wavenumber while the lowercase k denotes the site in the Kitaev chain. Setting $\mu_k = \mu$, $t_{jk} = t$ and $\Delta_{jk} = \Delta$ the following Hamiltonian is obtained

$$H = -\sum_K c_K^\dagger c_K \left(\mu + 2t\left(1 - \frac{1}{n}\right)\right)\cos(K) + \Delta\left(1 - \frac{1}{n}\right)\sum_K \left(e^{iK} c_{-K}c_K + e^{-iK} c_K^\dagger c_{-K}^\dagger \right). \tag{S5}$$

The entire procedure between Eq. (S4) to Eq. (S5) can be found for instance in Appendix B of Ref. [3]. When $n \to \infty$ the term $1 - 1/n = 1$ leading to the following Hamiltonian in K space

$$H = -\sum_K c_K^\dagger c_K (\mu + 2t)\cos(K) + \Delta\sum_K \left(e^{iK} c_{-KK} + e^{-iK} c_K^\dagger c_{-K}^\dagger \right). \tag{S6}$$

Going back to finite Kitaev chains and setting $t(1 - 1/n) = \tilde{t}$ and $\Delta(1 - 1/n) = \tilde{\Delta}$ in Eq. (S5) the same form of the Hamiltonian as in Eq. (S6) is obtained

$$H = -\sum_K c_K^\dagger c_K (\mu + 2\tilde{t})\cos(K) + \tilde{\Delta}\sum_K \left(e^{iK} c_{-K}c_K + e^{-iK} c_K^\dagger c_{-K}^\dagger \right). \tag{S7}$$

One can draw a conclusion that the behavior of the infinite Kitaev chains in K-space is the same as those of finite chains with a modified tunneling and superconducting pairing $t \to \tilde{t}$ and $\Delta \to \tilde{\Delta}$. When $\Delta = -t$ the topological condition in infinite Kitaev chains is $\mu < 2t$ [4, 5]. Given that topological properties are fully determined from properties of the K-space Hamiltonians [6] the topological condition for a finite Kitaev chain is

$$\mu < 2\tilde{t} = 2t\left(1 - \frac{1}{n}\right) \overset{n=3}{=} \frac{4}{3}t. \tag{S8}$$

The potentially topological state of matter in a finite Kitaev chain can be further described by a winding number ν [6]. Similarly with a Chern number the winding number is a topological invariant of a given system [5, 6]. In the case of the Kitaev chain, $\nu = \pm 1$ would indicate a topological phase and $\nu = 0$ a topologically trivial phase. The winding number is defined as [5, 6]

$$\nu = \frac{1}{2\pi}\int_{-\pi}^{\pi} dK \partial_K w(K), \tag{S9}$$

where $\partial_K w(K)$ is the winding number density and $w(K) = \mathrm{Arg}[2\tilde{\Delta}\sin(\mathrm{K}) + \mathrm{i}(\mu + 2\tilde{\mathrm{t}}\cos(\mathrm{K}))]$. Here Arg stands for a non-principal value of the argument. Note that the winding number is a $\mathbb{Z}$ topological invariant and it was long thought that it cannot be used to characterize Kitaev chains. Work of Ref. [7] changed this and proved the equivalence of Eq. (S9) and a more adapted expression for Kitaev chains. The integral in Eq. (S9) is difficult to compute due to the fact that the non-principal value of the argument cannot be found in an analytical fashion. Still, a numerical routine can be developed, by unwinding an array of different values of $w(K)$ in the region $[-\pi, \pi]$. In the results given at Fig. S1 we compare results of Eq. (S8) with a numerical solution of the integral in Eq. (S9) obtained by numerically unwinding $w(K)$ in the region $[-\pi, \pi]$ by Python's numpy unwind function.

In Fig. S1 we see that even the shortest possible Kitaev chain of only 2 sites, remains topological for $\mu < t = 1$ and that as the length of the chain is increased the topological-non-topological boundary approaches that of infinite chains $\mu < t = 2$ [4]. In the case of the experiment in the main body of the paper $n = 3$, $\mu_{cr} = 4/3$ and so the system remains topological in 75% of values of μ as compared to an infinite Kitaev chain. This means that the 9 points corresponding to low value of μ are in the topologically non-trivial regime in the main body of the paper.

Finally, two-qubit gates error rates of contemporary quantum computers ($< 0.75\%$) and are the most dominant source of errors on current-day quantum devices. We can try to estimate are such constrains on error rates of two qubit gates preventing one to achieve an MZM state on a quantum computer.

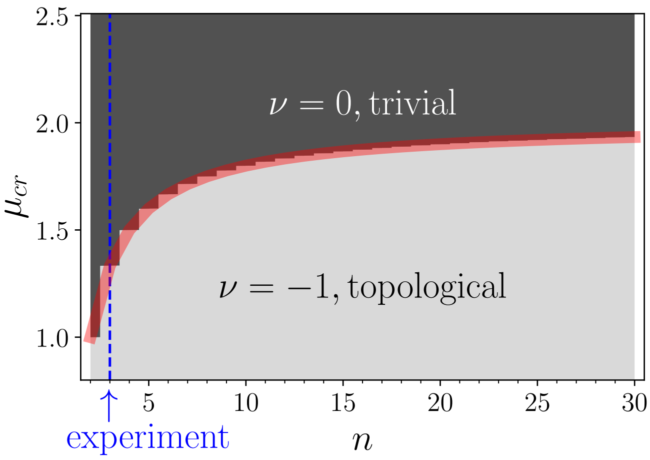

FIG. S1: The topological and non-topological region boundary as a function of Kitaev chain length. The figure shows the critical chemical potential which is the boundary between the two phases μ_{cr} when $t = -\Delta = 1$. The red line is the condition in Eq. (S8), while the grey areas are obtained by numerically unwinding and integrating Eq. (S9).

To measure that we define a parameter which represents a difference between the two-qubit gate angles at $\mu = 0^+$ and $\mu \approx 1.33$ normalized to π for every two qubit gate in the circuit preparing the ground state of the MZMs (Fig. 1 - main body of the paper)

$$\delta = \frac{\mathrm{abs}\left(\theta_{\mathrm{ij}}|_{\mu=0+} - \theta_{\mathrm{ij}}|_{\mu\approx 1.33}\right)}{\pi}. \tag{S10}$$

In Tab I we see that circuit is remaining in the topological regime even if the uncertainty in generating the two qubit gate is on the order of $\sim 0.75\%$ outlining the possibility of creating MZMs on modern gate-based quantum computers. The exception to this is the first two qubit gate between qubits q_1 and q_2 which has to have a value of $\pm\pi/2$ for a broad range of μ. However, the two-qubit $RYXXY$ gate has a special symmetry point at $\theta_{ij} = \pi/2$ in which it is equivalent to two single qubit gates Z-gates which have much lower error rates (on the order or $< 0.1\%$)

$$RYXXY\left(\frac{\pi}{2}\right) = e^{i\frac{\pi}{2}(\sigma_y^1\otimes\sigma_x^2 - \sigma_x^1\otimes\sigma_y^2)} = \sigma_z^1 \otimes \sigma_z^2. \tag{S11}$$

S3 - A NOISE MODEL

Noise in a quantum system governed by the Hamiltonian H and described by a density matrix ρ is given by

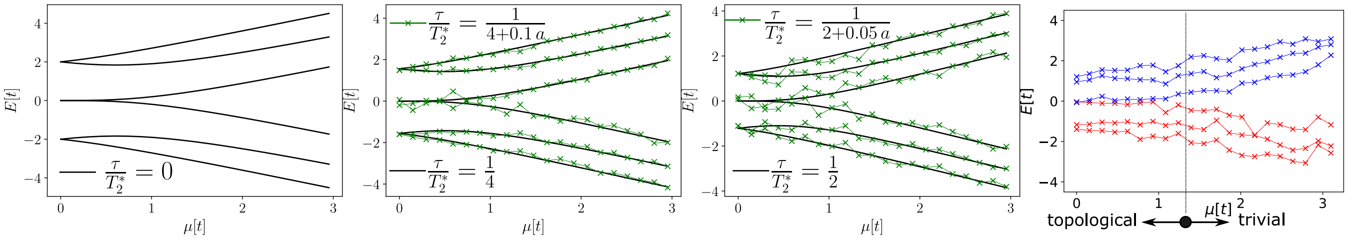

FIG. S2: First 3 figures - result of modeling, last figure - experimental data. a is a random number $a = [-1, 1]$

μ	$\theta_{12}(\pi)$	$\theta_{01}(\pi)$	$\theta_{12}(\pi)$	$\theta_{12}(\pi)$	$\theta_{01}(\pi)$	$\theta_{12}(\pi)$
0^+	0.500	0.304	0.333	0.333	0.304	0.250
0.155	0.500	0.317	0.337	0.314	0.301	0.257
0.310	0.500	0.327	0.342	0.294	0.298	0.264
0.465	0.500	0.334	0.347	0.272	0.294	0.273
0.620	−0.500	0.338	0.353	0.249	0.290	0.282
0.775	0.500	0.339	0.359	0.227	0.286	0.293
0.930	0.500	0.339	0.366	0.205	0.282	0.303
1.085	0.500	0.337	0.372	0.186	0.270	0.313
1.240	0.500	0.334	0.379	0.169	0.275	0.324
1.395	0.500	0.331	0.385	0.154	0.273	0.334
δ	0.0	0.9%	1.5%	5.2%	0.9%	2.4%

TABLE I: Angles of two qubit $RYXXY$ gates required to prepare Majorana zero modes - no color topological vs. yellow trivial. δ absolute difference between the two-qubit gates angle of the initial topological and trivial regions. The circuit has the same form like in the main body of the paper in Fig. 1.

[8]

$$\dot{\rho} = -\frac{i}{\hbar}[H, \rho] + \frac{1}{2}\sum_{ij}\Gamma_{ij}(2L_{ij}^{\dagger}\rho L_{ij} - L_{ij}L_{ij}^{\dagger}\rho - \rho L_{ij}L_{ij}^{\dagger}). \tag{S12}$$

Here, Γ_{ij} is the dissipation rate between the channel ij, and $L_{ij}^{\dagger} = |i\rangle\langle j|$ and $L_{ij} = |j\rangle\langle i|$ are Lindblad dissipation operators. In general if $i \neq j$ the terms in the Lindblad equation describe relaxation processes happening on a characteristic relaxation timescale T_1 and if $i = j$ these terms correspond to pure dephasing processes described by a characteristic time T_2^*. In general $T_2^* \ll T_1$ - pure dephasing dominates over relaxation - and this is the assumption I adopt in the remainder of the Section.

The solution of a Lindblad equation in the presence of only pure dephasing is

$$\tilde{\rho}(\tau) = \mathrm{diag}\left(\rho(0)\right) + \sum_{\mathrm{i}\neq\mathrm{j}}\rho_{\mathrm{ij}}\mathrm{e}^{-\frac{\tau}{\mathrm{T}_2^*}}, \tag{S13}$$

the diagonal part off the density matrix remains unchanged while the off-diagonal terms are decaying with a factor of $\exp\left(-\tau/T_2^*\right)$ (see Section "Phase damping" in Ref. [9]).

By repeating the procedure from the main body of the paper - obtaining the quantum circuit with OpenFermion and then getting the ground state wavefunction $|\psi\rangle$ in a form of a numerical vector we can obtain the density matrix $\rho(0) = |\psi\rangle\langle\psi|$. After obtaining the density matrix and multiplying its off-diagonal terms by $\exp\left(-\tau/T_2^*\right)$ we obtain the noisy density matrix of the system $\tilde{\rho}$ (this simulates idle noise processes). The noisy expectation value of the Hamiltonian can be obtained as

$$E = \mathrm{Tr}(\tilde{\rho}\,\mathrm{H}). \tag{S14}$$

Recent experimental and theoretical studies [10–13] indicate that the dephasing time of superconducting qubits varies in time. A minimal model in describing that is assigning random fluctuations of the dephasing time between measurements of different eigenstates of energy at different values of μ, $T_2^* \to T_2^* + ab$ where $a = [-1, 1]$ is a random number and $b \ll T_2^*$.

In Fig. S2 I show what is the effect of pure dephasing on the spectrum of MZMs. As the duration of the quantum circuit is increased, the circuit dephases more (first to third subfigure) the topologically trivial states ($E = \pm 2$ at $\mu = 0^+$) move more towards zero in the BdG spectrum (black lines). Furthermore, variations in the dephasing time between the realization of the experiment yield a qualitatively similar spectrum to the one measured (green symbols in the 3rd subfigure vs. measured data - 4th subfigure). This effect is visually similar to a slight violation of particle-hole symmetry. Noise in the quantum computing device could be understand as performing an MZM experiment with uncertainties in tunneling, chemical potential and superconducting pairing like that one would encounter in a realistic system.

S4 - THE MAJORANA EDGE CORRELATION FUNCTION

In the thermodynamic limit, the Majorana edge correlation function is expected to decay with a quadratic dependence in μ [14]. As comparing a $n = 3$ site Kitaev chain with a theory developed for an infinitely long chain

is infeasible, I will just give mean square fits of the decay of the absolute value of the Majorana edge correlation function $|\langle i\gamma_1\gamma_k\rangle|$, with a goal of outlining the fact that Majorana edge correlations do indeed decay as a function of μ. The polynomial fit to data for the first run (left) for state [0] is $0.57-0.18\mu+0.03\mu^2$ and for the state $[1,2]$ is $0.56-0.18\mu+0.03\mu^2$ with respective residuals 0.03 and 0.02.

The polynomial fit to data for the second run (right, main body of the paper) for state [0] is $0.63-0.14\mu+0.03\mu^2$ and for state $[1,2]$ $0.6-0.09\mu+0.01\mu^2$ with residuals of 0.01 and 0.03 respectively. The device was calibrated between runs.

S5 - COMPARISON BETWEEN RUNS OF HARDWARE

In this section I compare two different experiment runs on IBM Santiago in FIG. S3. Although the different executions vary slightly quantitatively depending on the calibration of the device all qualitative features remain present, especially the possible topological degeneracy. In Fig. S4 I display a comparison between a BdG spectrum and two different realizations of the experiment. Both experiments show excellent qualitative agreement with the BdG prediction. One can quantify this by defining a mean absolute error as $\mathrm{ME}=\left(\sum_i |x_i-y_i|\right)/m$, where m is the total number of measurements/predictions, x_i is the ith measurement of energy from the quantum device and y_i is the ith prediction of the BdG Hamiltonian. I find a ME $=0.127$ for the left figure and ME $=0.129$ for the right figure for $-1.87\leq y_i\leq 1.87$.

S6 - A 4-SITE KITAEV CHAIN

The formalism presented here allows the treatment of longer Kitaev chains with quantum simulators. Actual quantum computing preparation of Kitaev states should somewhat still be possible with currently available quantum computing hardware, however the author of this paper has no access to state of the art quantum computers and I will thus only focus on results from quantum simulators in the remainder of this Supplementary Material.

In FIG. S6 I compare results obtained by an ideal simulation of Gaussian states with BdG energies (a) and display the eigenspectrum obtained on a quantum computing simulator (b).

In subfigure (c) Majorana edge-correlation functions are shown with states [0] and $[1,2,3]$ having a decay of the edge correlation function. Parity switches are also in accordance with single-particle picture theories. In FIG. S7 I show a matching between parity switches predicted by Gaussian states implemented on an ideal quantum computing simulator and white lines are predictions of Eq. (2). Grey areas are ranges in parameter space of μ where parity switching exists due to a finite resolution of μ.

Also in similarity with the 3-site case Majorana zero modes start favoring pairing between neighboring Majoranas as the chemical potential is increased see states $[0,2,3]$ and [1] in FIG. S8.

* marko.rancic@totalenergies.com

[1] Z. Jiang, K. J. Sung, K. Kechedzhi, V. N. Smelyanskiy, and S. Boixo, Quantum algorithms to simulate many-body physics of correlated fermions, Phys. Rev. Applied **9**, 044036 (2018).
[2] J. R. McClean, N. C. Rubin, K. J. Sung, I. D. Kivlichan, X. Bonet-Monroig, Y. Cao, C. Dai, E. S. Fried, C. Gidney, B. Gimby, *et al.*, Openfermion: the electronic structure package for quantum computers, Quantum Science and Technology **5**, 034014 (2020).
[3] G. Skantzaris, *The conductivity of Kitaev's chain*, Master's thesis, Universiteit Utrecht (2014).
[4] A. Y. Kitaev, Unpaired majorana fermions in quantum wires, Physics-Uspekhi **44**, 131 (2001).
[5] N. Leumer, M. Marganska, B. Muralidharan, and M. Grifoni, Exact eigenvectors and eigenvalues of the finite kitaev chain and its topological properties, Journal of Physics: Condensed Matter **32**, 445502 (2020).
[6] C.-K. Chiu, J. C. Y. Teo, A. P. Schnyder, and S. Ryu, Classification of topological quantum matter with symmetries, Rev. Mod. Phys. **88**, 035005 (2016).
[7] S. Tewari and J. D. Sau, Topological invariants for spin-orbit coupled superconductor nanowires, Phys. Rev. Lett. **109**, 150408 (2012).
[8] M. J. Rančić and G. Burkard, Electric dipole spin resonance in systems with a valley-dependent g factor, Phys. Rev. B **93**, 205433 (2016).
[9] M. A. Nielsen and I. Chuang, Quantum computation and quantum information (2002).
[10] P. V. Klimov, J. Kelly, Z. Chen, M. Neeley, A. Megrant, B. Burkett, R. Barends, K. Arya, B. Chiaro, Y. Chen, A. Dunsworth, A. Fowler, B. Foxen, C. Gidney, M. Giustina, R. Graff, T. Huang, E. Jeffrey, E. Lucero, J. Y. Mutus, O. Naaman, C. Neill, C. Quintana, P. Roushan, D. Sank, A. Vainsencher, J. Wenner, T. C. White, S. Boixo, R. Babbush, V. N. Smelyanskiy, H. Neven, and J. M. Martinis, Fluctuations of energy-relaxation times in superconducting qubits, Phys. Rev. Lett. **121**, 090502 (2018).
[11] S. Schlör, J. Lisenfeld, C. Müller, A. Bilmes, A. Schneider, D. P. Pappas, A. V. Ustinov, and M. Weides, Correlating decoherence in transmon qubits: Low frequency noise by single fluctuators, Phys. Rev. Lett. **123**, 190502 (2019).
[12] J. J. Burnett, A. Bengtsson, M. Scigliuzzo, D. Niepce, M. Kudra, P. Delsing, and J. Bylander, Decoherence benchmarking of superconducting qubits, npj Quantum Information **5**, 1 (2019).
[13] J. Etxezarreta Martinez, P. Fuentes, P. Crespo, and J. Garcia-Frias, Time-varying quantum channel models

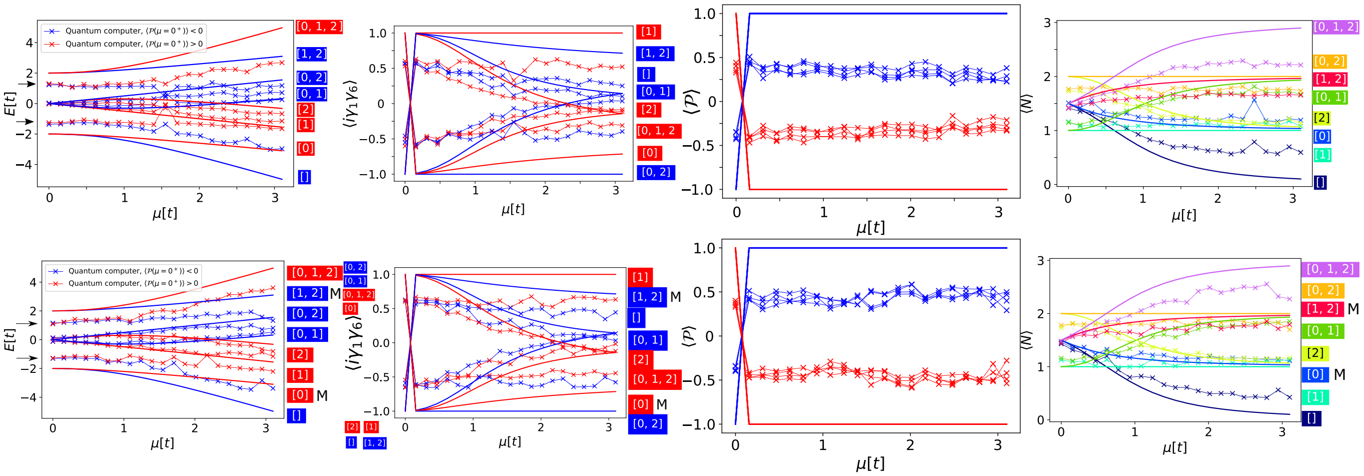

FIG. S3: TOP - first realisation of: Energy, Majorana edge correlation function, parity and particle number respectively as a function of chemical potential. BOTTOM - second realisation (main body of paper) of: Energy, Majorana edge correlation function, parity and particle number respectively as a function of chemical potential. Full lines denote ideal simulation, x-points connected with lines are a result of an experiment on IBM Santiago.

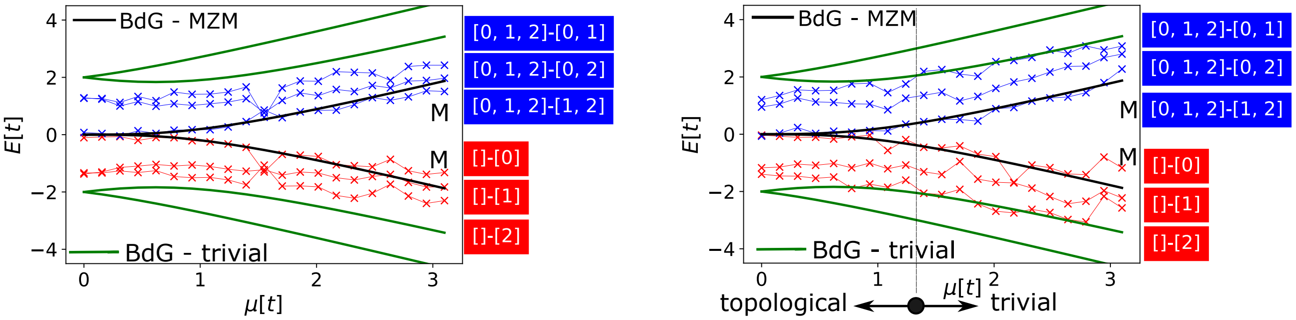

FIG. S4: The BdG Hamiltonian theory (black and green) and experiment (blue and red). LEFT first realisation of the experiment, RIGHT second realisation of the experiment (main body of the paper).

for superconducting qubits, npj Quantum Information **7**, 1 (2021).

[14] J.-J. Miao, H.-K. Jin, F.-C. Zhang, and Y. Zhou, Majorana zero modes and long range edge correlation in interacting kitaev chains: analytic solutions and density-matrix-renormalization-group study, Scientific reports **8**, 1 (2018).

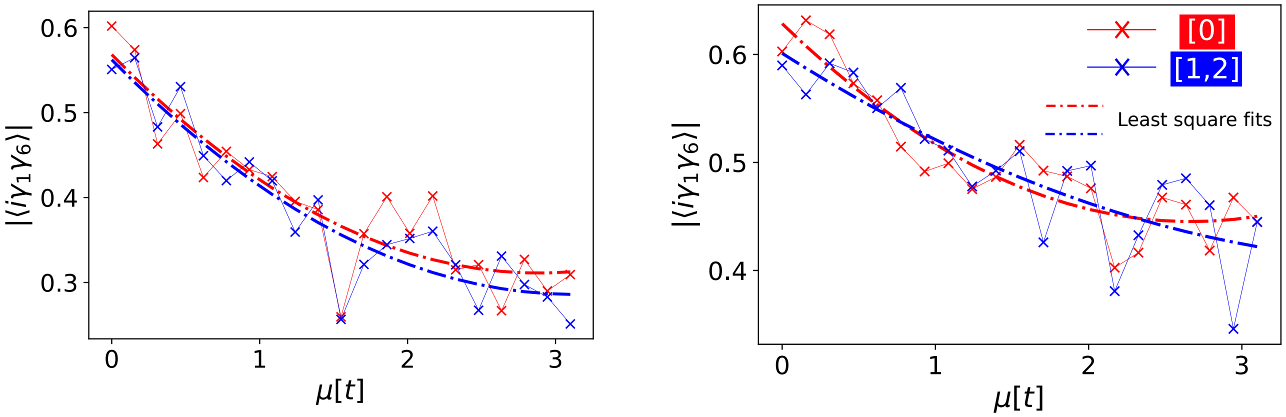

FIG. S5: The absolute value of the correlation function of Majorana zero modes. LEFT first realization of the experiment, RIGHT second realization of the experiment (main body of the paper).

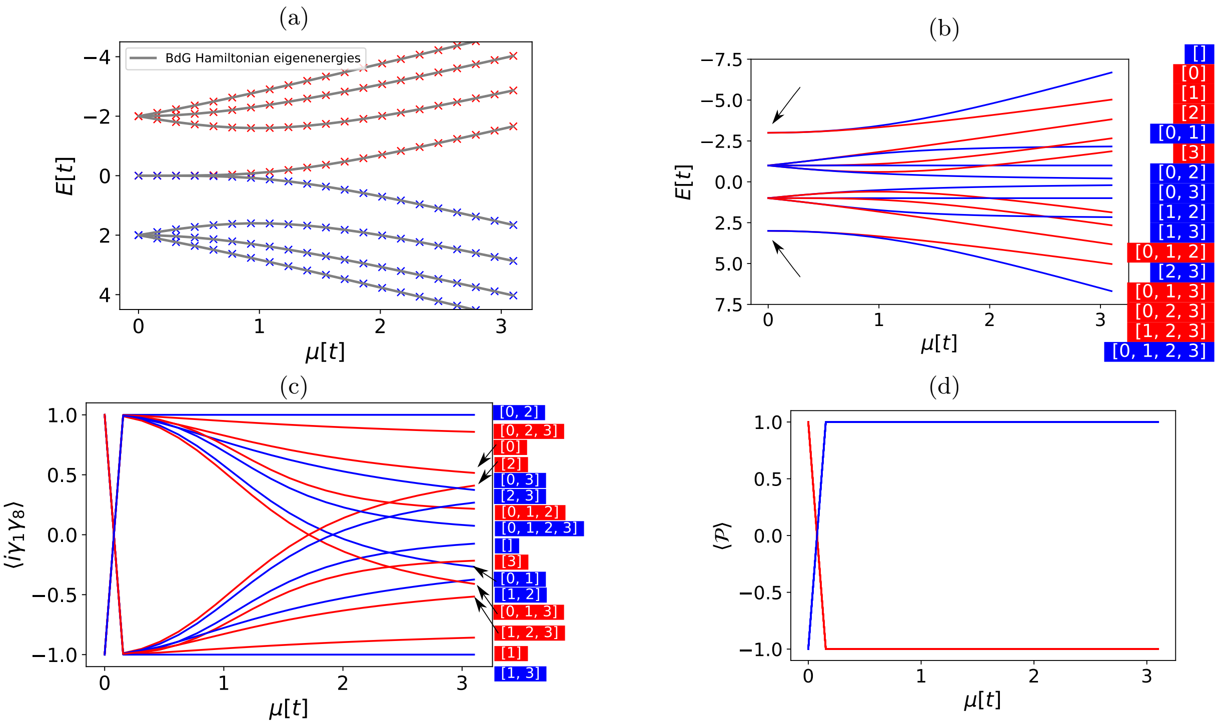

FIG. S6: Quantities of a 4-site Kitaev Hamiltonian at $t = -1$ and $\Delta = 1$ as a function of the chemical potential μ in units of absolute value of tunnelling $[t]$. Red denotes values obtained on an ideal simulator of quantum computers with $\langle P(\mu = 0^+)\rangle = 1$. Blue denotes values obtained on an ideal simulator of quantum computers with $\langle P(\mu = 0^+)\rangle = -1$. (a) The single-particle picture BdG spectrum, diagonalizatioin of the BdG Hamiltonian (grey lines) compared to Gaussian state solutions (coloured markers). (b) The full spectrum computed by Gaussian states. Black arrows indicate the topological degeneracy. (c) Majorana edge-correlation function, an ideal simulation with fermionic Gaussian states implemented on a quantum computing simulator. (d) Parity - an ideal simulation with Gaussian states.

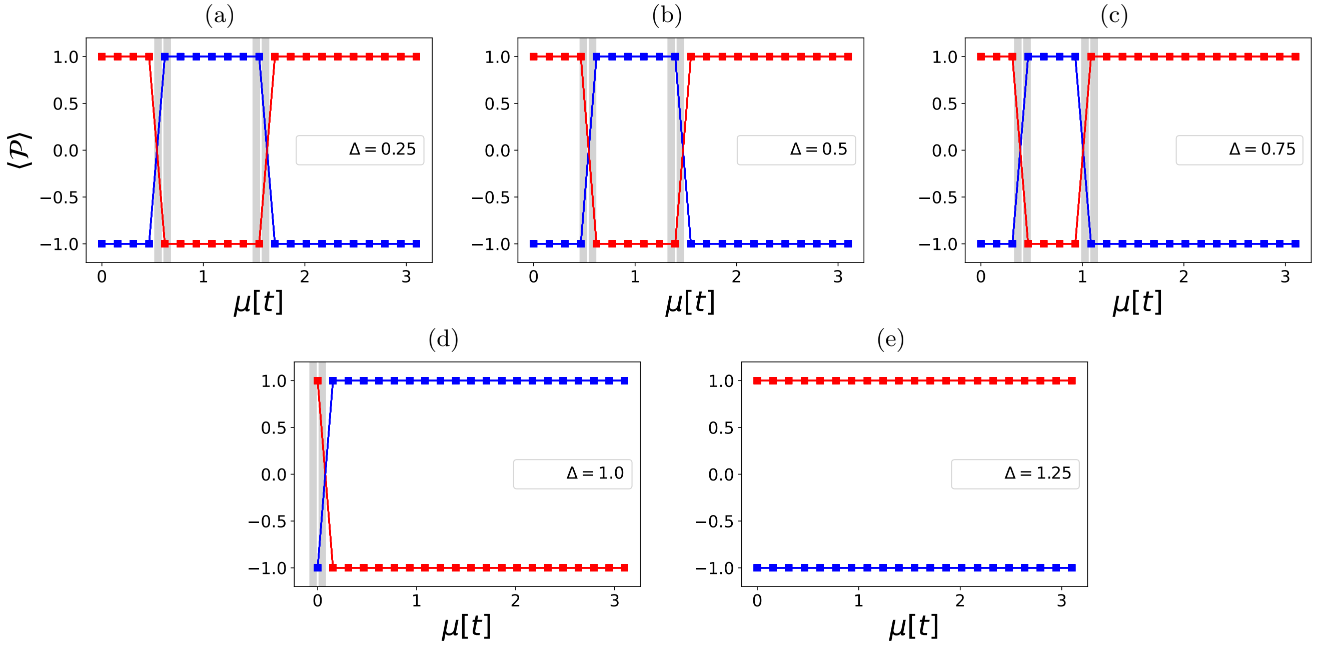

FIG. S7: (a-e) Parity switches at $t = -1$ for different values of Δ and for 20 values of μ between 10^{-8} and 3.1 with a 0.155 increment for an $n = 4$ Kitaev chain. White lines are predictions of Eq. (2) and grey areas are the regions in which parity switches potentially exist due to finite resolution in μ.

.

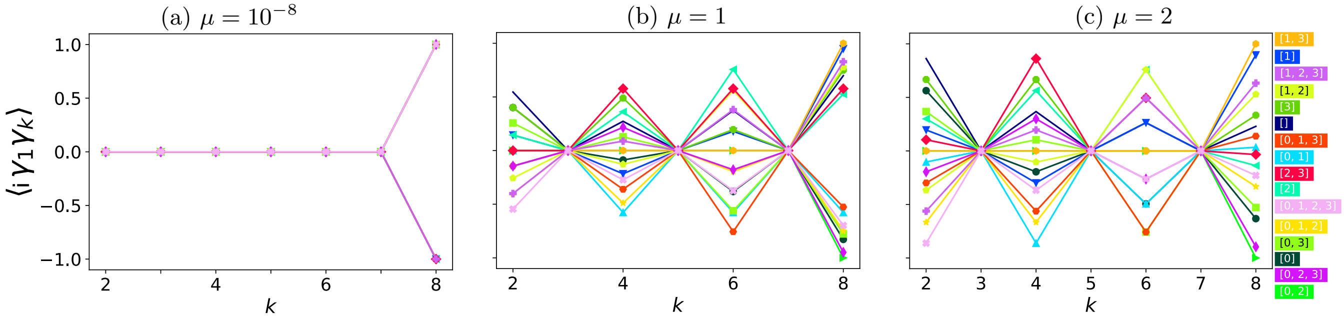

FIG. S8: Site correlation function where k denotes the site at different values of the chemical potential in the topological regime $\Delta = -t = 1$ obtained by fermionic Gaussian states. The colour code is explained on the right.
